# Transcription factor TFIIEβ interacts with two exposed positions in helix 2 of the Antennapedia homeodomain to control homeotic function in *Drosophila*

**DOI:** 10.1371/journal.pone.0205905

**Published:** 2018-10-15

**Authors:** Claudia Altamirano-Torres, Jannet E. Salinas-Hernández, Diana L. Cárdenas-Chávez, Cristina Rodríguez-Padilla, Diana Reséndez-Pérez

**Affiliations:** 1 Department of Immunology and Virology, College of Biological Sciences, Universidad Autónoma de Nuevo León, San Nicolás de los Garza, Nuevo León, México; 2 Department of Cell Biology and Genetics, College of Biological Sciences, Universidad Autónoma de Nuevo León, San Nicolás de los Garza, Nuevo León, México; University of Dayton, UNITED STATES

## Abstract

Homeoproteins contain the conserved homeodomain (HD) and have an important role determining embryo body plan during development. HDs increase their DNA-binding specificity by interacting with additional cofactors outlining a Hox interactome with a multiplicity of protein-protein interactions. In *Drosophila*, the first link of functional contact with a general transcription factor (GTF) was found between Antennapedia (Antp) and BIP2 (TFIID complex). Hox proteins also interact with other components of Pol II machinery such as the subunit Med19 from Mediator (MED) complex, TFIIEβ and transcription-pausing factor M1BP. All these interactions clearly demonstrate Hox-driven transcriptional regulation, but the precise molecular mechanism remains unclear. In this paper, we focused on the Antp-TFIIEβ protein-protein interface to establish the specific contacts as well as its functional role. Using Bimolecular Fluorescence Complementation (BiFC) in cell culture and *in vivo* we found that TFIIEβ interacts with Antp through the HD independently of the YPWM motif and the direct physical interaction is at helix 2, specifically aminoacidic positions I32 and H36 of Antp. We also found, through ectopic assays, that these two positions in helix 2 are crucial for Antp homeotic function in head involution, and thoracic and antenna-to tarsus transformations. Interestingly, overexpression of Antp and TFIIEβ in the antennal disc showed that this interaction is required for the antenna-to-tarsus transformation. In conclusion, interaction of Antp with TFIIEβ is important for the functional specificity of Antennapedia, and amino acids 32 and 36 in Antp HD helix 2 are key for this interaction. Our results open the possibility to more broadly analyze Antp-TFIIEβ interaction on the transcriptional control for the activation and/or repression of target genes in the Hox interactome during *Drosophila* development.

## Introduction

Hox proteins specify the segmental identity along the body’s antero-posterior axis and have a pivotal role in animal morphogenesis, development and evolution [1–3]. They also control many cellular functions such as cell migration, shape, proliferation, apoptosis and differentiation [4,5]. These proteins contain the homeodomain (HD) that allows DNA recognition, determining tissue specificity by regulating expression of target genes [6]. Since HDs are highly conserved with overlapping DNA binding properties, it is unclear how Hox proteins instruct differential morphogenesis postulating the so called “Hox specificity paradox” [7]. To provide a plausible solution for the specificity functions *in vivo*, it has been shown that Hox proteins, through different regions, bind to DNA with additional cofactors and can also interact with transcriptional regulatory factors, non-coding RNAs, cell matrix proteins and chromatin remodeling complexes [8,9], outlining a Hox interactome with a multiplicity of protein-protein interactions. In these interactions, the relationship between Hox proteins and the general transcription machinery is very important for transcriptional regulation. The first evidence of a Hox protein establishing functional contact with a General Transcription Factor (GTF) is the interaction between the YPWM motif of Antennapedia (Antp) and BIP2 (TAF3), a TATA-binding protein-associated component of TFIID [10]. Additionally, the Med19 subunit of the MED complex, the major component of Pol II machinery, interacts with Proboscipedia (Pb), Deformed (Dfd), Antp, Ultrabithorax (Ubx), Abdominal A (AbdA) and Abdominal B (AbdB) [11]. Hox proteins are also involved in the mechanisms of pausing Pol II, interacting with the transcription-pausing factor M1BP, changing the chromatin state and enhancing transcription [12]. A Hox interactome screening of 35 transcription factors found that TFIIEβ interacts with Scr, Antp, Ubx, AbdA and AbdB in *Drosophila* embryo [13]. All these interactions clearly demonstrate Hox-driven transcription, but the precise molecular mechanisms of transcriptional regulation remain elusive. Since transcriptional regulation has been considered to happen mainly on the transcription preinitiation complex (PIC), we focused on the protein-protein interface of Antp-TFIIEβ to establish the specific contacts on Antp, as well as the functional role of this interaction. Our results showed a direct physical interaction of TFIIEβ with two aminoacidic positions of Antp HD at helix 2, and this interplay was absolutely required for the ectopic function of Antp in thorax and antenna-to-tarsus transformations. Our results provide insights into the molecular mechanisms of Hox protein action and extend the perspectives to explore activation and/or repression of target genes.

## Results

### TFIIEβ interacts with Antp through the HD

**Fig 1:**
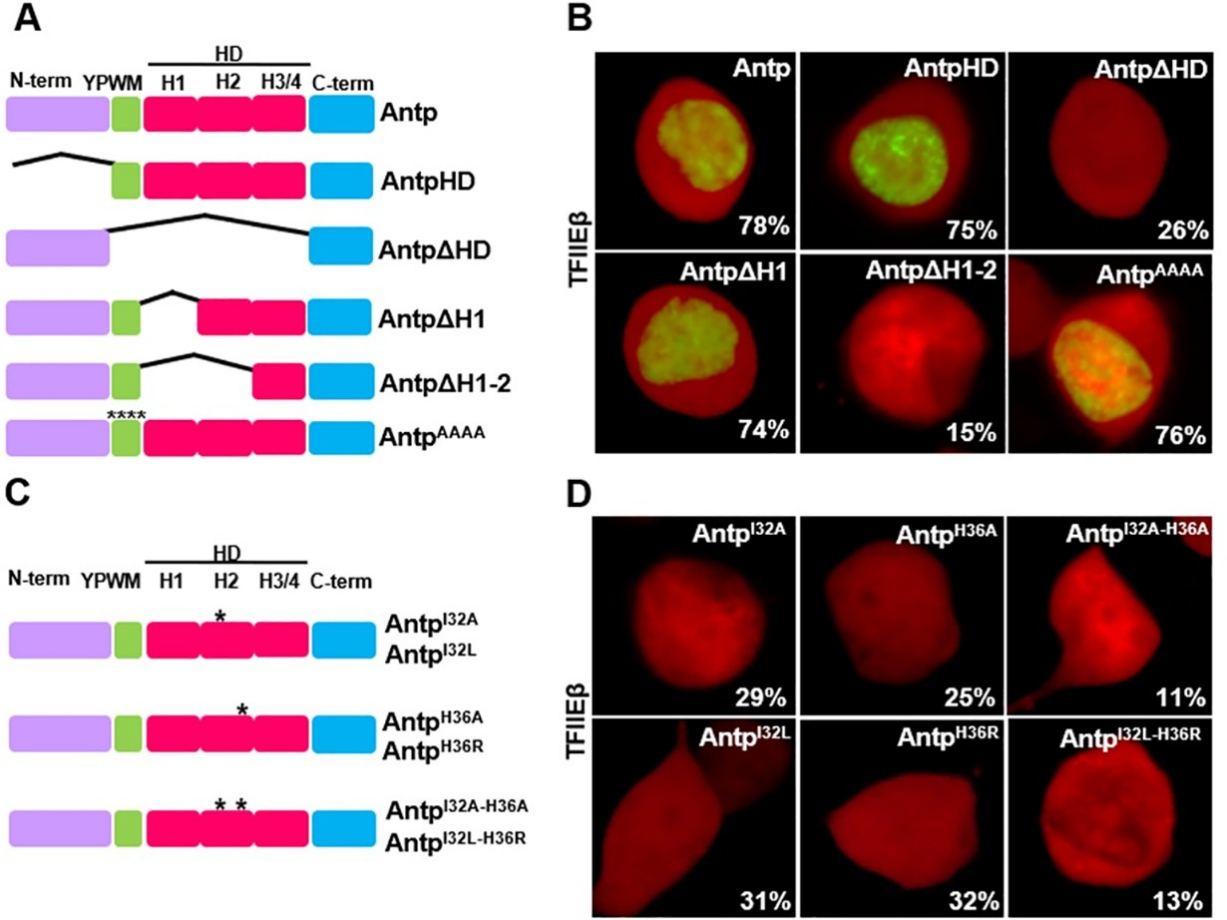
Antp HD helix 2 is required for interaction with TFIIEβ. (A) Schematic representation of the Antp constructs in pCS2-VC155 vector using the split Venus fragments (VC and VN). Antp domains, HD deletions in helices 1 and 2 and YPWM substitutions to alanines are indicated (drawn to scale). (B) BiFC assays showed protein-protein interaction between Antp and TFIIEβ (Venus reconstitution in green) which is dependent of the presence of Antp HD helix 2. (C) Schematic representations of single (*) or double (**) site-directed mutagenesis of helix 2 residues 32 and 36. (D) BiFC assays showed a drastic reduction of the interaction between TFIIEβ and Antp mutants indicating the interaction depends specifically on aminoacidic positions 32 and 36. pCAG-Cherry was co-transfected as a control (red fluorescence).

To identify the region of Antp involved in the interaction with TFIIEβ, we performed a series of deletions and site-directed mutagenesis to determine its interaction by Bimolecular Fluorescence Complementation (BiFC) in cell culture. Deletion of the HD (AntpΔHD) decreased dramatically the interaction with TFIIEβ; only 26% of the AntpΔHD cells showed a highly significant difference in interaction compared to 78% of the cells with wild-type Antp and 75% with AntpHD (Fig 1A and 1B; S1 Fig). We also replaced the Antp YPWM motif with alanines (Antp^AAAA^), showing not effect on the interaction (76%). These results indicate that Antp HD is necessary and the YPWM motif is not required for Antp-TFIIEβ interaction in transfected cells.

### Residues 32 and 36 at Antp HD helix 2 are essential for interaction with TFIIEβ *in vivo*

**Fig 2:**
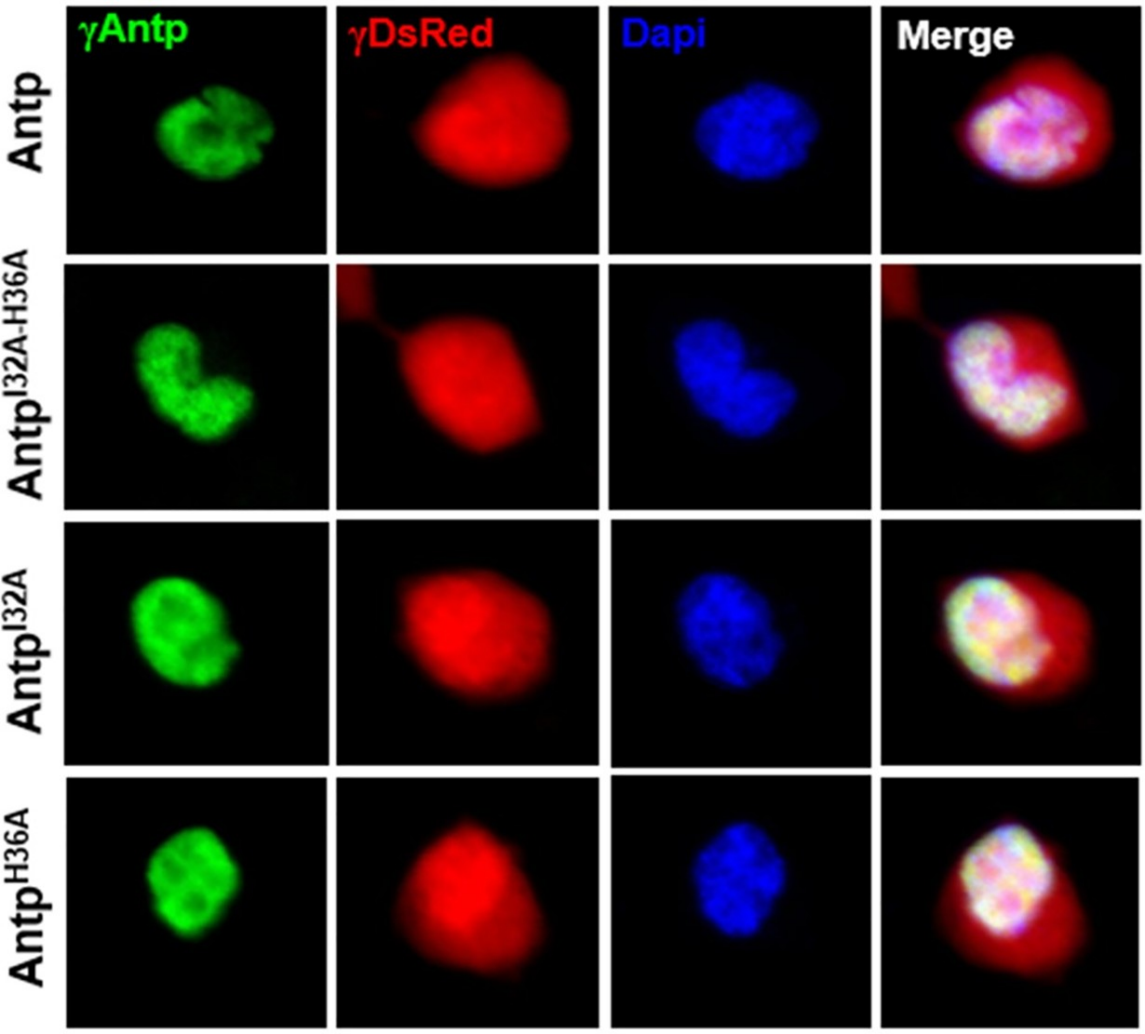
Antp helix 2 mutants localize in the nucleus of HEK293 cells. Fluorescence immunostaining in transfected cells using γAntp4C3 (green) colocalize with nuclei staining with Dapi (blue) demonstrating the nuclear localization of Antp and Antp helix 2 mutants.

Since Antp-TFIIEβ interaction was dependent on the HD, we deleted helix 1 of the HD (AntpΔH1) as well as helices 1 and 2 (AntpΔH1-2). Deletion of helix 1 maintained the interaction with TFIIEβ (74%) while deletion of helices 1 and 2 significantly reduced the interaction to 15% (Fig 1 and S1 Fig). These results suggested that helix 2 of Antp HD is responsible for this interaction. Further, we analyzed exposed positions on helix 2 opposite to the HD-DNA binding that are conserved within other *Drosophila* Hox proteins. Helix 2 aminoacidic residues I32 and H36 were substituted to alanine (I32A, H36A) or to structurally similar leucine and arginine (I32L, H36R). We generated single HD mutants: Antp^I32A^, Antp^H36A^, Antp^I32L^ and Antp^H36R^ as well as double mutant: Antp^I32A-H36A^ and Antp^I32L-H36R^ by site-directed mutagenesis (Fig 1C). Co-transfection of Antp helix 2 single mutants and TFIIEβ-expressing vectors showed a reduction of the interaction ranging from 25% to 36% (Fig 1D). Interaction of Antp helix 2 double mutants with TFIIEβ showed that simultaneous mutagenesis of these residues caused a drastic reduction of the interaction, to 11% with alanine substitutions and 13% with leucine and arginine substitutions. All TFIIEβ interactions with single and double helix 2 mutants showed a highly significant difference compared to interactions with Antp (S1 Fig). In addition, inmunostaining of Antp on the transfected cells clearly demonstrated the nuclear localization of helix 2 Antp mutants confirming that the loss of interaction obtained is not due to the mis-localization of the mutant proteins (Fig 2). These results indicate that I32 and H36 at helix 2 of HD are directly involved on Antp-TFIIEβ interaction in cell culture.

**Fig 3:**
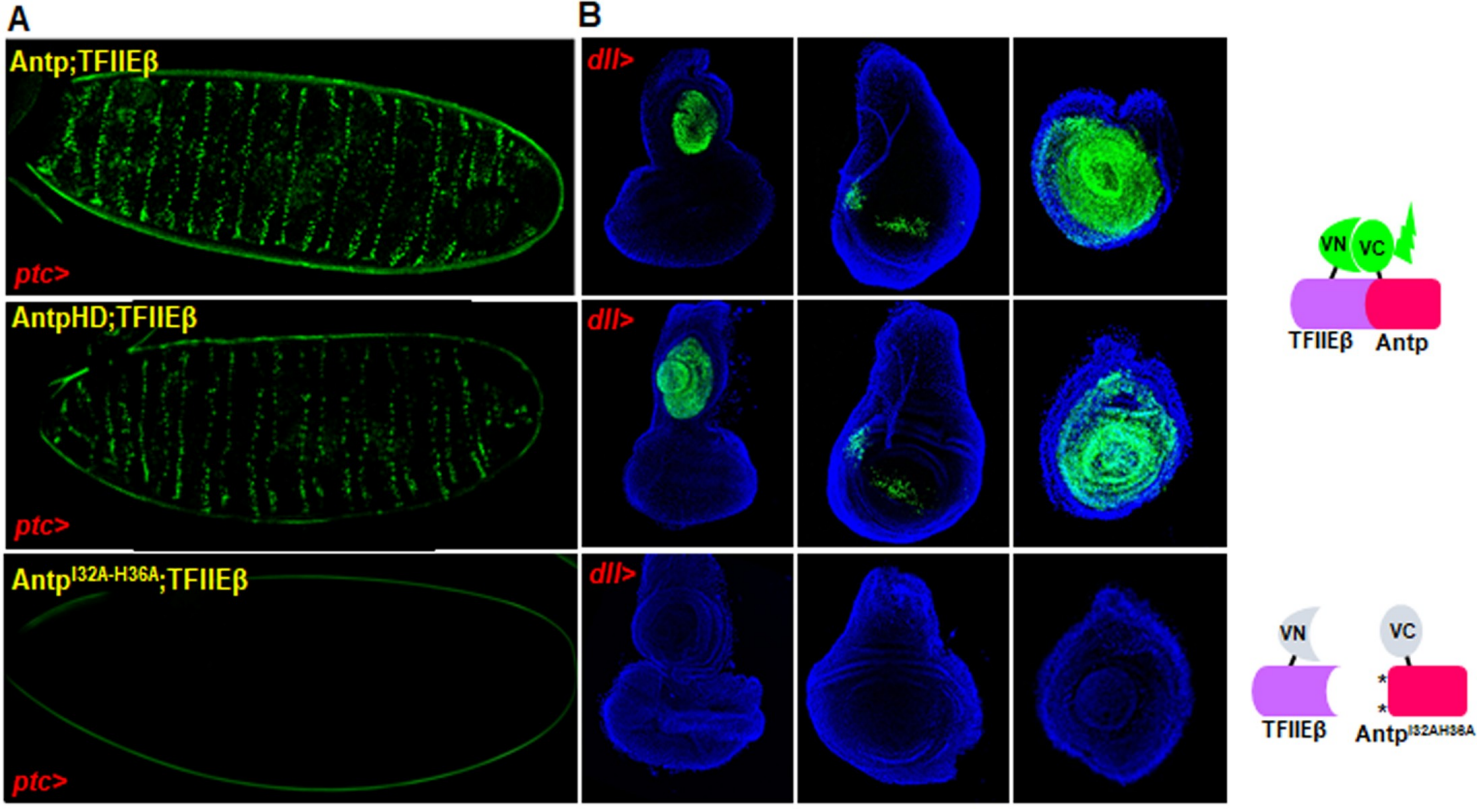
Residues 32 and 36 of Antp HD helix 2 are essential for interaction with TFIIEβ *in vivo*. (A) Antp full length and HD interactions with TFIIEβ were shown by BiFC in embryos, whereas no interaction (BiFC signal) was detected with Antp helix 2 double mutant. (B) In imaginal discs, Antp-TFIIEβ interactions were detected in the eye-antenna, wing and leg discs (upper and middle panels), in contrast no interaction was detected with Antp helix 2 double mutant (lower panel). Imaginal discs were dyed with Dapi (blue) for structure visualization.

Moreover, to confirm the requirement of residues 32 and 36 on the AntpHD-TFIIEβ interaction obtained in cell culture, we choose the double alanine helix 2 Antp mutant to perform *in vivo* BiFC assays on both *Drosophila* embryos and imaginal discs. UAS reporter flies bearing VC-Antp, VC-AntpHD or Antp^I32A-H36A^ along with VN-TFIIEβ were crossed with *ptc* or *dll*- drivers for embryo and imaginal disc expression, respectively. Both Antp and AntpHD interactions with TFIIEβ (BiFC signal) were detected in embryos and imaginal discs (Fig 3A and 3B upper and middle panels). In contrast, when we expressed the VC-Antp^I32A-H36A^ double mutant along with VN-TFIIEβ we did not detect significantly different BiFC signals neither in embryos nor in the imaginal discs (Fig 3A and 3B lower panel and S2 Fig), indicating that the interaction with TFIIEβ depends on Antp HD helix 2 residues. We also detected that expression of the YPWM mutant and TFIIEβ showed a reduction of the signal interaction in embryos as shown in S3 Fig.

**Fig 4:**
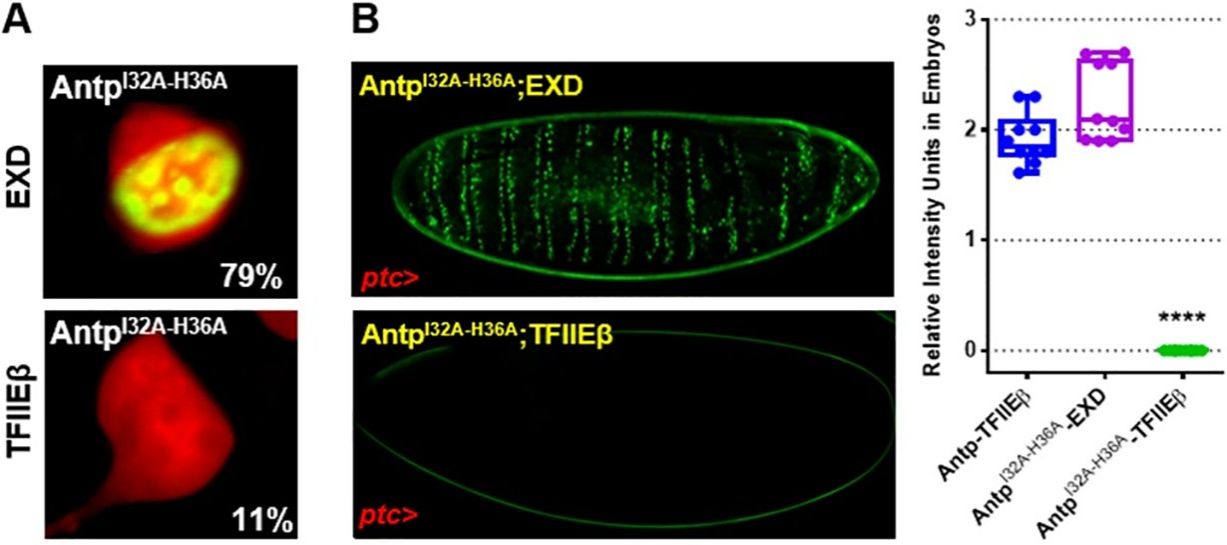
Double mutant Antp^I32A-H36A^ interacts with EXD. (A) BiFC assays in HEK293 cells showed 79% of protein-protein interaction between double mutant Antp^I32A-H36A^ and EXD (Venus reconstitution in green) in contrast with the lack of interaction with TFIIEβ (11%). (B) Antp^I32A-H36A^ interacts with EXD in embryos, but not with TFIIEβ. Relative intensity units quantification of BiFC interaction was done using the color histogram function of ImageJ in embryos. We used one-way ANOVA and the post-hoc test Tukey for mean comparison. There is no statistical difference between Antp-TFIIEβ and Antp^I32A-H36A^-EXD interactions. Errors bars correspond to standard deviation.

Additionally, we found that the helix 2 mutant activates transcription using BS2 binding sites (S4 Fig) and we included a control co-expressing EXD with Antp double mutant to provide evidence that helix 2 mutation is not disrupting protein conformation. Results showed a very clear interaction between EXD and Antp^I32A-H36A^ mutant in cells and embryos, indicating that this mutation is not affecting Antp interaction with EXD (Fig 4). We also verified that double Antp mutant protein presented similar expression levels compared to Antp and TFIIEβ (S5 Fig).

Our results showed for the first time that the interaction between Antp and TFIIEβ is dependent of Antp HD through residues 32 and 36 at helix 2 in embryos and imaginal discs.

### Antp HD residues 32 and 36 are required for homeotic function in *Drosophila*

**Fig 5:**
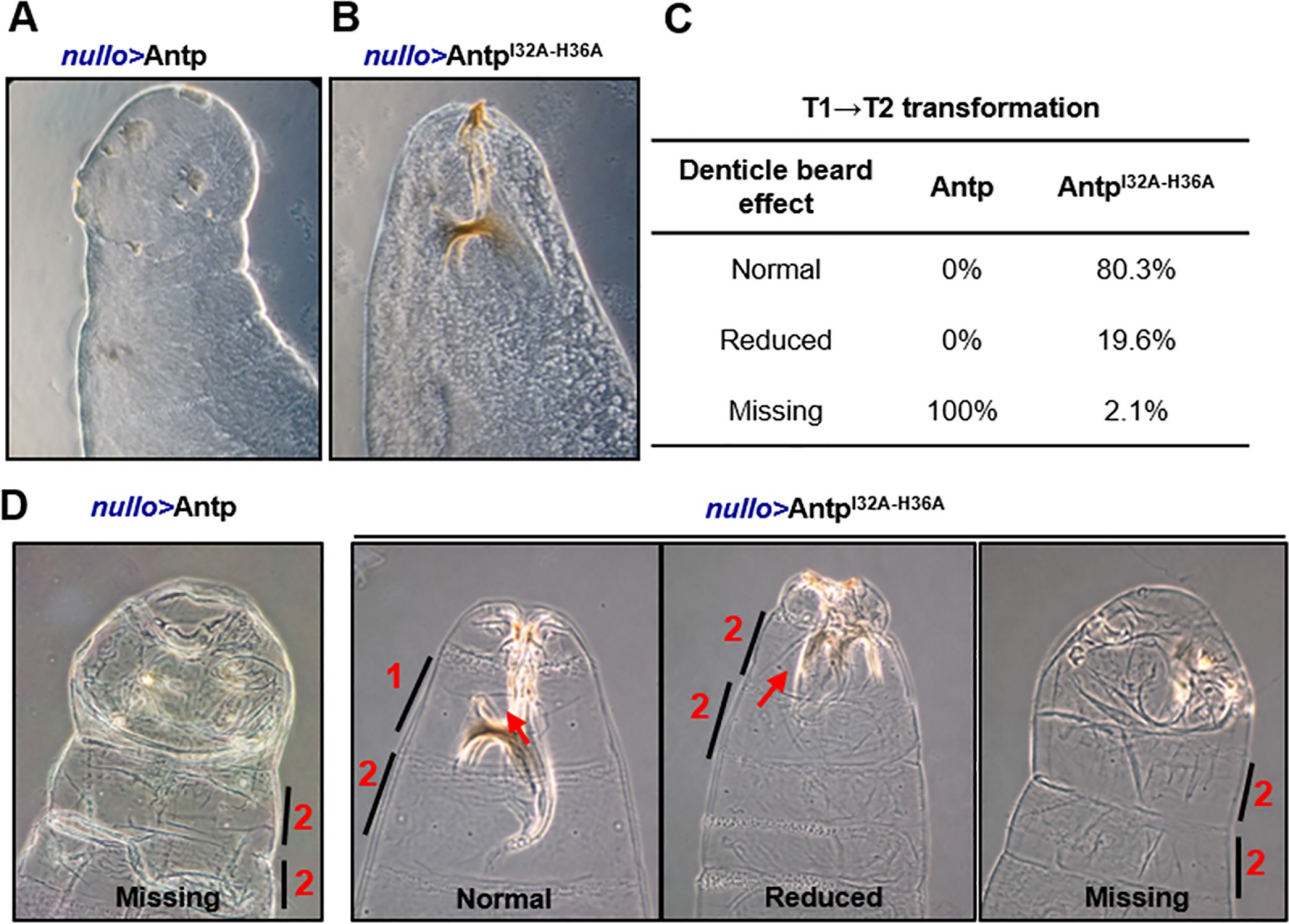
Antp^I32A-H36A^ failed to inhibit head involution and homeotic transformation in embryos. (A) Embryo cuticle showing head-involution inhibition and transformation of prothoracic segment (T1) into a mesothoracic segment (T2) caused by Antp wt expression. (B) Embryo cuticle with ectopic mutant expression does not affect head-involution showing wild type phenotype. (C) Most of the Antp^I32A-H36A^ embryos had “normal” T1 identity (80%) compared to Antp wt embryos that showed all “missing” prothoracic denticle beards indicating T1-to-T2 homeotic transformation (100%). (D) The numbers 1–2 and lines show the corresponding segment identity: prothoracic (1) or mesothoracic (2), and red arrows show the prothoracic beards.

To determine the functional importance of Antp interaction with TFIIEβ, we tested whether the double mutant Antp^I32A-H36A^ showed the functional transformation in an ectopic assay. Overexpression of the double mutant Antp^I32A-H36A^ driven by *nullo* did not affect head involution, showing a normal head phenotype (Fig 5B), and a normal T1 denticle beard (80%) on most of the embryos (Fig 5C and 5D). As expected, Antp caused complete inhibition of head involution (Fig 5A) and missing denticle beard with transformed prothoracic segment T1 into T2 in all embryos (Fig 5C and 5D). The lack of transformation of Antp^I32A-H36A^ double mutant expression indicated that residues 32 and 36 of HD helix 2 are required for the function of Antp in *Drosophila*.

**Fig 6:**
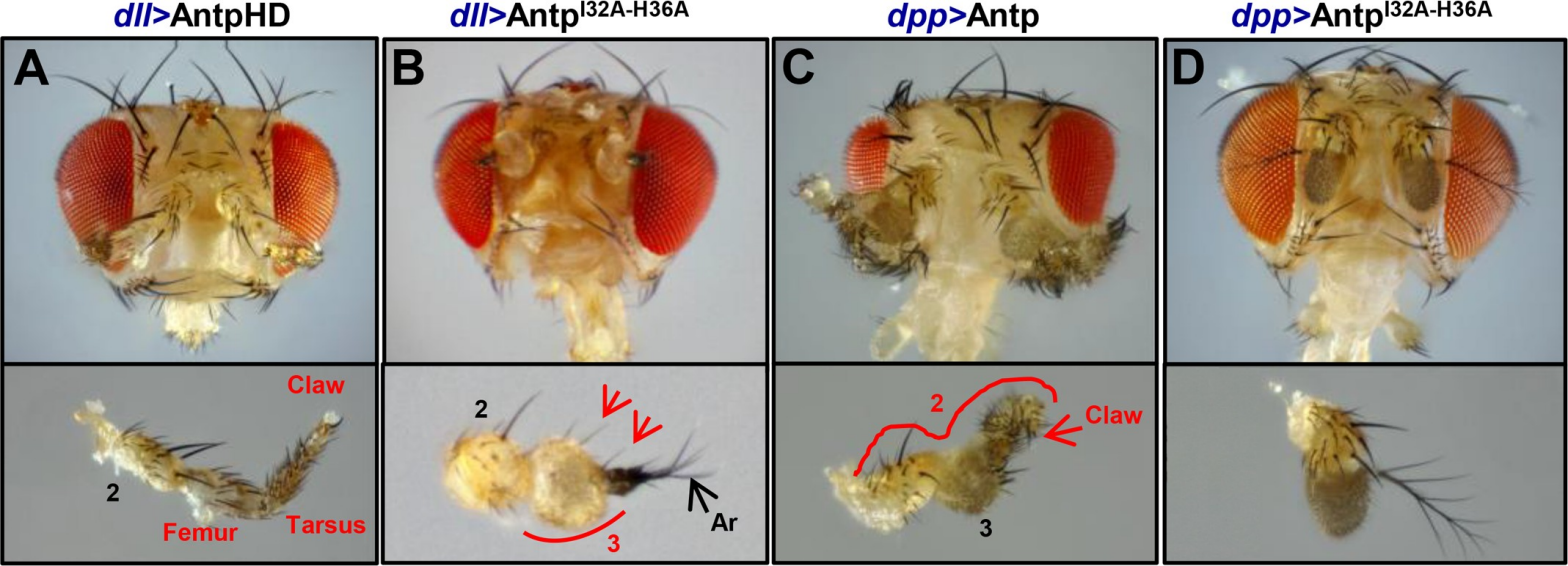
Ectopic expression of Antp^I32A-H36A^ reduced antenna-to-tarsus transformation in adult heads. (A) The AntpHD expression in the antenna showed its transformation into a mesothoracic leg (T2) identity as it is clearly appreciated in the dissected antenna. Note the presence of tarsal claws that identify strong transformations (lower panel). (B) Double mutant Antp^I32A-H36A^ expression showed a drastic reduction of the transformation effect. Note the presence of leg bristles (arrowhead) indicative of a weak transformation of antennal tissue to leg fate (lower panel). (C) Antp expression driven by *dpp*-Gal4 caused the transformation of antennal segment 2 into T2 leg tissue. (D) Mutant Antp^I32A-H36A^ expression showed no transformation. Transformed segments are identified using red color and curves; 2, second antennal segment; 3, third antennal segment; Ar, arista.

We also analyzed the functional homeotic transformation of double mutant Antp^I32A-H36A^ using *dll* and *dpp* drivers to generate antenna-to-tarsus transformations. Directed expression of double mutant Antp^I32A-H36A^ to the third antennal segment suppresses the homeotic transformation (Fig 6B and S1 Table); we observed only a thickening of the arista and third antennal segment (A3) with presence of scattered ectopic leg bristles (Fig 6B, arrowheads) which are indicative of a weak transformation of antennal tissue to leg fate. AntpHD showed the ectopic transformation of the antenna into T2 mesothoracic leg featuring coxa, femur, tibia and tarsal structures such as the claw showing a strong transformation (Fig 6A and S1 Table); single expression of TFIIEβ directed to the antenna does not show any transformation (S1 Table).

The same was true for expression of the Antp double mutant on the second antennal segment, with inhibition of the homeotic transformation on the antenna; all the segments appeared totally normal (Fig 6D). Meanwhile, the expression of Antp showed a strong homeotic transformation of the antenna as shown in Fig 6C. These results demonstrate that residues 32 and 36 of Antp HD helix 2 are required for Antp homeotic function in antenna-to-tarsus transformation.

### AntpHD-TFIIEβ interaction is crucial for the antenna-to-tarsus transformation

**Fig 7:**
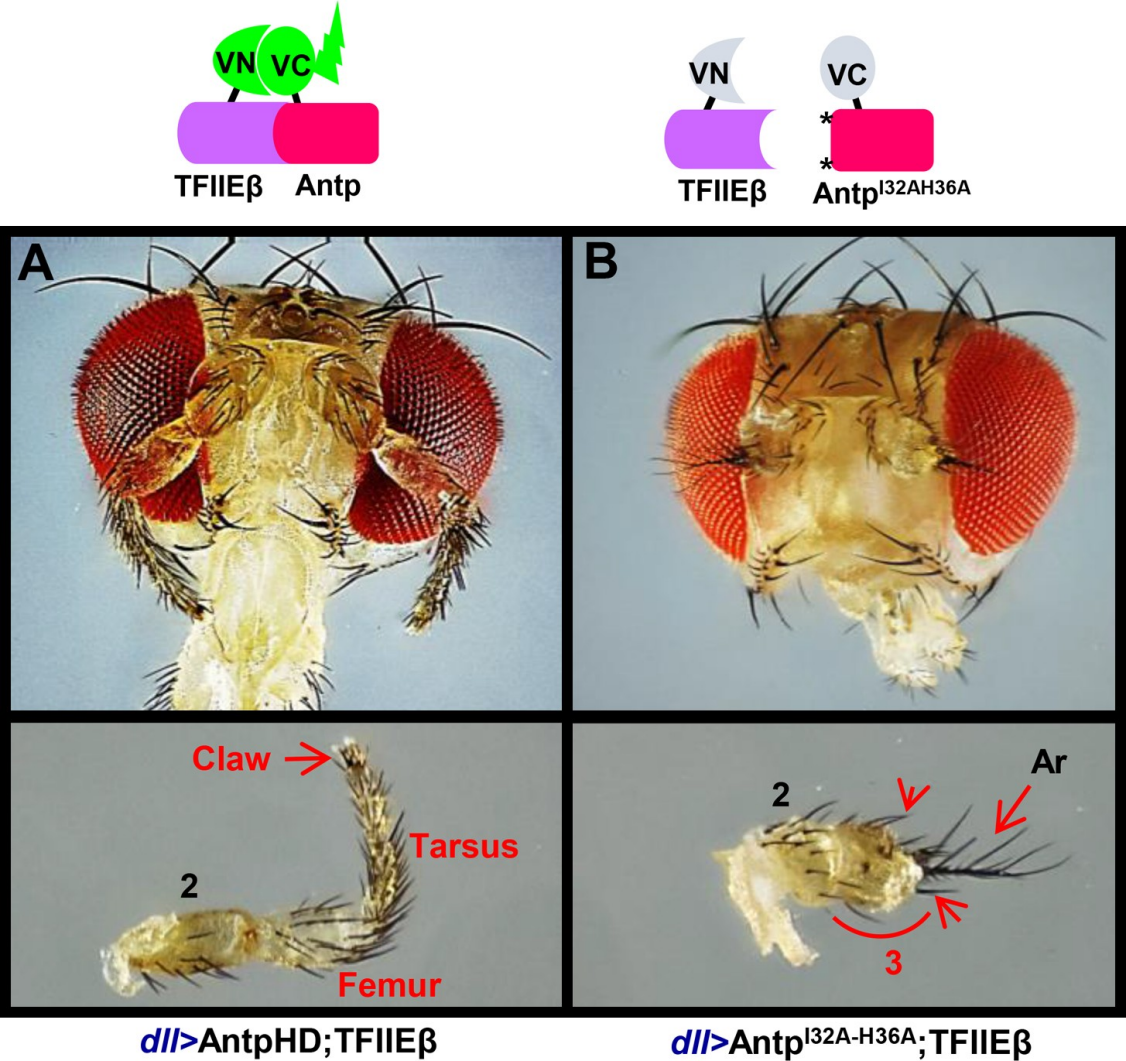
AntpHD-TFIIEβ interaction is crucial for antenna transformation in adult flies. (A) The ectopic expression of AntpHD and TFIIEβ showed a strong antenna transformation toward T2 leg tissue in adults obtained from larvae containing BiFC interaction signal (AntpHD-TFIIEβ). Note the presence of tarsal claws (lower panel). (B) The co-expression of TFIIEβ and Antp^I32A-H36A^ double mutant in the antenna showed a drastic reduction of the transformation. Note the 3rd antennal segment only barely overgrown, mild arista thickening and the presence of leg bristles indicative of a weak transformation (arrowheads). Transformed segments are identified using the color red and curves; 2, second antennal segment; 3, third antennal segment; Ar, arista.

To approach the functional relevance of the Antp-TFIIEβ interaction, we directed expression of VC-Antp^I32A-H36A^ along with VN-TFIIEβ using *dll*. We selected larvae that contained the BiFC interaction signal and let them develop into adults to be sure of the presence of the interacting proteins. The co-expression of AntpHD and TFIIEβ showed a very strong homeotic transformation of the antenna into T2 mesothoracic leg tissue (Fig 7A). Co-expression of TFIIEβ and double mutant Antp^I32A-H36A^, however, showed a normal antenna with a very faint transformation: the antenna showed a barely overgrown 3rd antennal segment, mild arista thickening and several ectopic leg bristles (Fig 7B, lower panel arrowheads). The drastic reduction of the antenna transformation is attributable to the lack of interaction between the double mutant and TFIIEβ, thus confirming that this interaction is necessary for the homeotic antenna-to-tarsus transformation in *Drosophila*.

## Discussion

To analyze the interplay between Hox and the general transcription machinery, we focused on Antp-TFIIEβ protein-protein interface to establish the specific contacts, as well as the functional role of this interaction. Our results showed a direct physical interaction of TFIIEβ with the 32 and 36 positions of helix 2 Antp HD in cell culture and *in vivo*. These two positions on helix 2 HD are required for interaction with TFIIEβ, and this interaction is necessary for homeotic transformation.

Our results demonstrate that Antp HD was necessary for maintaining the interaction with TFIIEβ. Previous studies have confirmed that the HD is sufficient for interaction with GTFs. For example, Baeza *et al*. [13] and Hudry *et al*. [14] found that the AbdA HD was sufficient for TFIIEβ interaction and that when the DNA-binding of the HD is mutated, the interaction is diminished but not abolished. Another example came from Boube *et al*. [11], who used BiFC *in vivo* to demonstrate that the Ubx HD and AbdA HD are sufficient for direct interaction with Med19. In addition of the conserved HD affinities to DNA and RNA [6,15], several protein-protein interactions also relied on the HD, such as dimerization of Scr, and Antp interaction with Eyeless [16–17].

Although we found that Antp-TFIIEβ interaction is YPWM-independent in BiFC cell culture and the presence of an intact YPWM motif in the helix 2 Antp mutant showed neither interaction by BiFC nor functional activity, co-expression of the YPWM mutant and TFIIEβ reduced the signal interaction in embryos as shown in S3 Fig. A similar result in embryos was found by Baeza *et al*. [13] where YPWM Antp mutant showed a reduction but not an abolition of TFIIEβ interaction on *Drosophila* embryos, that could be attributable to the presence of helix 2 in the mutant. Altogether, this suggest that interactions of Antp with TFIIEβ could change from one tissue to another with complex formation in different tissues using various interfaces (YPWM and/or HD), contributing to the plasticity of Hox interaction properties.

Deletional analysis of Antp HD suggested interaction of TFIIEβ through the helix 2 of Antp HD. Based on the reported 3D-structure of Antp HD DNA complex [6], in which helix 2 is on the opposite side of the HD-DNA binding, we selected the conserved residues 32 and 36, which are exposed and physically available, as candidates for TFIIEβ interaction. To perform a molecular dissection on the Antp-TFIIEβ interaction, we analyzed the residues I32 and H36 of helix 2, either individually or together, by site-directed mutagenesis in cell culture. BiFC results show a drastic reduction of the interaction by mutation of these two residues, indicating that they are directly involved on Antp-TFIIEβ interaction. It has been demonstrated that AntpHD is internalized to the nuclei [18], through the residues 43–58 of the third helix [19]. Therefore, since our mutations are present on helix 2, the Antp NLS were not affected. To confirm that, we performed immunostaining of Antp helix 2 mutants on cells and embryos showing very clearly the nuclear localization of Antp helix 2 single mutants and double mutant Antp (Fig 2). These results indicated that Antp helix 2 mutants include NLSs for their localization into the nucleus. Moreover, we also demonstrated that helix 2 mutant keeps its transactivation activity (S4 Fig) and is capable to interact with EXD in cells and embryos (Fig 4) confirming that mutation of these amino acids did not alter DNA binding affinity and the protein conformation to perform essential activities required for *in vivo* transformation.

Since both substitutions by alanines or structurally similar residues affected Antp-TFIIEβ interaction in cell culture in the same manner, we selected I32A-H36A HD mutant for the *in vivo* analysis in *Drosophila*. In concordance with BiFC cell culture assay, our results showed no interaction in embryos or in imaginal discs with Antp mutant I32A-H36A. Therefore, residues 32 and 36 of Antp helix 2 are crucial for the interaction with TFIIEβ in BiFC assays in *Drosophila* embryos and imaginal discs. This is relevant because residues 32 and 36 on Antp helix 2 are identical and highly conserved within *Drosophila* Hox proteins and can be extrapolated for the interaction with TFIIEβ to another homeoproteins due to the high Hox conservation.

Although our results very clearly show Antp-TFIIEβ interaction through positions 32 and 36 of helix 2, this does not exclude the possibility of another aminoacidic positions, either at helix 2 or the intervening loop, that could be involved to a minor extent on the interaction. For example, position 30 and 33, in addition to the helix 2 amino acids 32 and 36, have also been reported in human POU proteins Oct-1 and Oct-2 interaction with VP16 transactivator factor of Herpes Simplex Virus [20–21].

Because the precise molecular mechanisms of Antp in transcriptional regulation remains unclear, we attempted to shed light on these by determining whether I32 and H36 are important for Antp function. When Antp is ectopically expressed on embryos it causes inhibition of head-involution and transformation of prothoracic segment T1 into T2 and antennae into mesothoracic (T2) legs [22]. Antp ectopic expression exhibits that residues 32 and 36 of HD helix 2 are essential for its function in embryo head involution and homeotic transformations of thorax and antenna. Lack of homeotic transformations of Antp^I32A-H36A^ double mutant expression indicates that residues 32 and 36 of HD helix 2 are absolutely required for the Antp ectopic homeotic function in *Drosophila*. Likewise, Antp mutated in the YPWM motif is not capable of transforming the antenna [9], and a single exposed residue on helix 1 of Scr HD is necessary for its homeotic function [16], showing that beside the HD DNA-binding, exposed positions on the HD are crucial for Hox functional activity.

To determine the functional relevance of the Antp-TFIIEβ interaction, we directed co-expression of TFIIEβ and double mutant Antp^I32A-H36A^ to the antenna, showing a drastic reduction of the antenna transformation. These findings clearly demonstrate that Antp-TFIIEβ interaction (visualized by BiFC in live larvae) is necessary for the Antp homeotic function with a very strong transformation of the antenna into T2 mesothoracic leg. Together, our results imply that very subtle changes of two amino acids in the Antp HD helix 2 can have dramatic effects on protein-protein interaction with TFIIEβ, affecting transcriptional control and the functional properties of antenna-to-tarsus transformation.

These results show that the interaction between TFIIEβ and Antp HD contributes to transcriptional regulation and functional activities of Antennapedia. In the Pol II PIC formation, TFIIE is a heterodimer with α and β subunits, regulating TFIIH activities such as kinase on RNA Pol II CTD, ATPase [23] and DNA helicase [24]. TFIIEβ binds to both TFIIB and TFIIF in important activities needed for promoter melting and stabilization as well as for the transition to elongation. Thus, Antp-TFIIEβ interaction may represent a key control point for modulation of transcription factors involved in activation or repression functions. Repression activity of Antp-TFIIEβ interaction may imply destabilization of the PIC complex or the inhibition of TFIIEβ functions modulating TFIIH ATPase, CTD kinase or helicase activities. For example, it has been determined by *in vitro* transcription and co-immunoprecipitation assays that the zinc-finger TF Krüppel (Kr), a *Drosophila* segmentation protein for late embryonic development, interacts in a dimeric way with TFIIEβ and this interaction represses transcription [25]. If we consider that Antp dictates leg fate by repressing the activity of antenna-determining genes such as *Hth* and *Dll* in the leg imaginal discs [26–27], it could be reasonable that Antp-TFIIEβ can be involved in repression. Co-expression of Antp with TFIIEβ resulted in a reduction to 47% of the expression of Luciferase compared with of Antp alone (S4 Fig), however further experiments need to be done to evaluate the precise molecular mechanism of this interaction. It could also be possible that Antp facilitates the arrival of TFIIEβ to the PIC and subsequently the recruitment and/or activation of TFIIH, allowing an efficient transcription elongation. For example, mutation of *Med19* on haltere imaginal discs shows that Med19 is required for Ubx target gene activation [11]. Another example would be that Kr binds to TFIIB in a monomeric way, and this interaction activates transcription *in vitro* [25]. Thus, further experiments are needed to determine the fine molecular mechanism of how interaction between Antp and TFIIEβ contribute to transcriptional regulation by activation or repression activities, or even both.

Here, we presented a clear interaction of TFIIEβ with two aminoacidic positions of Antp HD that are important for Antp homeotic function, and this interplay is essential to the Antp antenna-to-tarsus transformation. In conclusion, amino acids 32 and 36 of Antp HD helix 2 play a very important role in determining the specificity of the TFIIEβ interaction. Altogether, these results provide insights into the molecular interface of Antp HD with TFIIEβ to evaluate the extent to which these molecular contacts translate into functional properties in activation or repression of target genes. The role of residues 32 and 36 on Antp helix 2 can be extrapolated for the interaction of TFIIEβ with other homeoproteins, for example Scr, Ubx and AbdA [13–14,28], due to the highly Hox conservation. In addition, Antp-TFIIEB interaction open the possibility to more broadly explore the interplay between Antp and additional transcription factors in the Hox interactome for the genetic control of development in *Drosophila*.

## Materials and methods

### Plasmid constructs

For BiFC assays, Antp, AntpHD, Antp^AAAA^ and AntpΔHD coding sequences were amplified by PCR from pNPAC constructs and cloned next to the C-terminal (VC) fragment of Venus into pCS2VC155. AntpΔH1 and AntpΔH1-2 deletions were obtained by site-directed mutagenesis (Quickchange II XL kit, Stratagene, La Jolla, CA, USA). The single and double mutants: Antp^I32A^, Antp^H36A^, Antp^I32H^, Antp^H36R^, Antp^I32A-H36A^ and Antp^I32H-H36R^ were also obtained by site-directed mutagenesis in the pCS2VC155-Antp vector as a template. For VNTFIIEβ fusion protein, TFIIEβ coding sequence was amplified by PCR from pET3-TFIIEβ donated by [29] and cloned next to the N-terminal (VN) fragment of Venus in the pCS2VNm9 vector. For the microinjection vectors, the DNA fragments coding for Antp^I32A-H36A^ and VCAntp^I32A-H36A^ were amplified by PCR and subcloned via NotI restriction site into pUASTattB. pNPACAntp^I32A-H36A^ transactivation vector was obtained by subcloning from pUASTattB via NotI. Plasmid constructs were verified by DNA sequencing before cell transfections or fly microinjection.

### Fly strains

Fly stocks and crosses were incubated either at 25°C or 18°C on standard yeast-agar-cornmeal medium. UAS-VCAntp^I32A-H36A^ and UAS-Antp^I32A-H36A^ transgenic lines were generated by ФC-32 integrase transformation; the Antp^I32A-H36A^ or VCAntp^I32A-H36A^ constructs were introduced into the attP landing site 86Fb on the third chromosome (M{3xP3-RFP-attP}ZH-86Fb strain). Other fly lines used were kindly donated: UAS-VCAntp, UAS-VNTFIIEβ, UAS-VNEXD and UAS-VCAntpHX by Samir Merabet [14], UAS-VCAntpHD by Yoshi Adachi, or obtained from Bloomington stock center: *nullo*-GAL4, *dpp*-GAL4, *ptc*-GAL4, *dll*-Gal4 and UAS-Antp.

### BiFC and transactivation assays in cell culture

HEK293 cells were cultured in MEM (GIBCO, Carlsbad, CA, USA) supplemented with 10% FBS (Invitrogen, Carlsbad, CA. USA) and 1% penicillin-streptomycin (Sigma-Aldrich, Saint Louis, MI, USA). The cells were plated on 6-well plates with glass coverslips and cultured for 24 h after which DNA constructs were transfected by using Polietilenimine (PEI) 15 nM (Sigma-Aldrich, Saint Louis, MI, USA), according to the manufacturer’s instructions. All BiFC co-transfections included the plasmid pCAG-mCherry (donated by Ataúlfo Martínez-Torres) for the calculation of transfection efficiency as internal control and transactivation assays used pcopia-βGal for normalization of luciferase expression. The BiFC and red fluorescence signals were visualized 48 h after transfection by mounting the cell coated coverslips onto slides on an Olympus BX61W1 confocal microscope and the Fluoview 4.0a software (Olympus, Tokyo, Japan). To exclude interference from autofluorescence, microscopy analysis was standardized with untransfected and transfected cells using the variable barrier filter function of Olympus Fluoview 4.0a software that restricts the specific wavelengths of the Venus emission spectra (514–527 nm). The percentage of the BiFC interactions was analyzed by counting the number of Venus fluorescent cells in one hundred red fluorescent cells (mCherry) of three independent experiments. In transactivation assays, luminescence was determined 48 h after transfection using the Dual-Luciferase Reporter Assay System Kit (Promega, Madison, WI, USA) according to the manufacturer’s instructions.

### BiFC assays in *Drosophila*

Fly crosses were incubated at 25°C overnight to visualize BiFC interaction complexes according to [14]. Embryos were dechorionated with 1.5% sodium hypoclorite, washed with PBX buffer and mounted on slides with 60% Glycerol/PBS. Imaginal discs were dissected on PBS and mounted on slides using Vectashield mounting medium with Dapi (Vector laboratories, Southfield, MI, USA).

### Embryo and adult cuticle preparation

Embryo cuticle preparations were carried out according to [30] and mounted on slides with a 10% KOH solution. For adult imaging, the heads and antennae were dissected and directly transferred to microscopic slides without coverslips. An average of 60 photographs were taken for each head and 30 for each antenna on Axioscope 40 microscope (Zeiss, Oberkochen, Germany). The images were merged using the software HeliconFocus version 6.

### Cells and embryo immunostaining

Cell culture and embryo immunostaining were performed according to standard procedures. Antibodies used were chicken yGFP (Abcam ab13970, 1:800), mouse yAntp4C3 (Developmental Studies Hybridoma Bank, University of Iowa 1:50) and gout yDsRed (Santa Cruz Biotechnology, 1:200).

## Supporting information

S1 Table Quantification of antenna transformations. (TIF)

S1 Fig Statistical analysis of Antp-TFIIEβ interactions in HEK293 cells by BiFC. The percentage of the BiFC interactions was analyzed by counting the number of Venus fluorescent cells in one hundred red fluorescent cells (mCherry) of three independent experiments. Statistical quantifications of Antp-TFIIEβ interactions were analyzed using a one-way ANOVA and the post-hoc test Tukey for mean comparison. Antp interaction with TFIIEβ was compared with Antp mutants. AntpHD, AntpΔH1 and Antp^AAAA^ interactions showed no difference compared to Antp, in contrast there is a highly significant difference (****) between Antp and the mutants AntpΔHD, AntpΔH1-2, as well as all helix 2 mutants (p < 0.005). Error bars correspond to standard deviation.
(TIF)

S2 Fig Statistical analysis of Antp-TFIIEβ interactions in embryos and imaginal discs by BiFC. Relative intensity units quantification of BiFC interactions was done using the color histogram function of ImageJ in embryos (A) and in imaginal discs (B). For the statistical analysis we used a one-way ANOVA and the post-hoc test Tukey for mean comparison (p < 0.005). Error bars correspond to standard deviation.
(TIF)

S3 Fig Mutation of the YPWM motif affects Antp-TFIIEβ interaction in *Drosophila* embryos. (A) Interactions of Antp, AntpHD and Antp^AAAA^ with TFIIEβ were showed in embryos using BiFC, meanwhile no interaction (BiFC signal) was detected with Antp helix 2 double mutant. (B) Relative intensity units quantification of BiFC interactions in embryos showed significant difference between Antp^AAAA^ interaction with TFIIEβ (**) compared to Antp or AntpHD (p < 0.005). Error bars correspond to standard deviation.
(TIF)

S4 Fig Antp^I32A-H36A^ activates transcription using BS2 binding sites. (A) Schematic representation pPAC plasmids containing coding sequences for Antp, AntpΔHD and Antp^I32A-H36A^ directed with Actin5C promoter and luciferase reporter (LUC) containing a minimal Hsp70 promoter and eleven tandem copies of HD-consensus BS2 binding sites. (B) Co-expression of Antp with TFIIEβ resulted in a reduction of 47% (****) of the expression of LUC compared with transactivation of Antp alone (p < 0.005). Mutation of the HD (AntpΔHD) show a 33-fold decrease of transcription (****), whereas the double mutant Antp^I32A-H36A^ activates transcription at the same level of Antp through the BS2 binding sites (p < 0.005). Error bars correspond to standard deviation.
(TIF)

S5 Fig Expression levels of Antp and TFIIEβ by fluorescence immunostaining. (A) Immunodetection of VCAntp, VCAntp^I32A-H36A^ and Antp^I32A-H36A^ was done using γAntp4C3 and VNTFIIEβ using γ-GFP. (B) Expression level of Antp and TFIIEβ were quantified using the color histogram function of ImageJ and compared to Antp. Relative intensity Units were measured from 5 embryos per construct line using the color histogram function of ImageJ. All lines exhibited expression levels equal or lower than the endogenous Antp protein. Error bars correspond to standard deviation.
(TIF)
