# Supplementary figures and images for: Transcription factor TFIIEβ interacts with two exposed positions in helix 2 of the Antennapedia homeodomain to control homeotic function in Drosophila
Source: PLoS One. 2018 Oct 15;13(10):e0205905. doi: 10.1371/journal.pone.0205905 (PMC6188894; doi:10.1371/journal.pone.0205905)

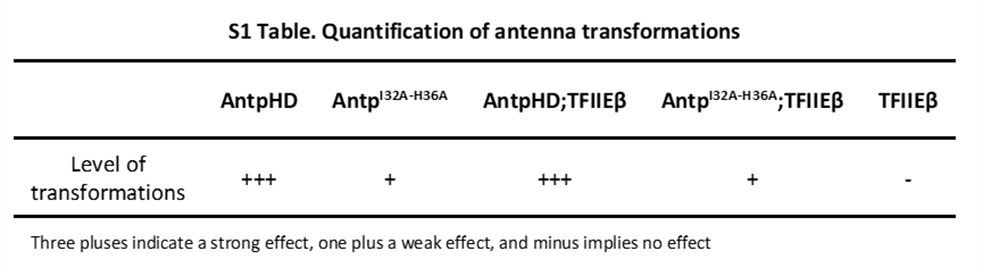

Supplement: S1 Table — (TIF) [file pone.0205905.s001.tif]

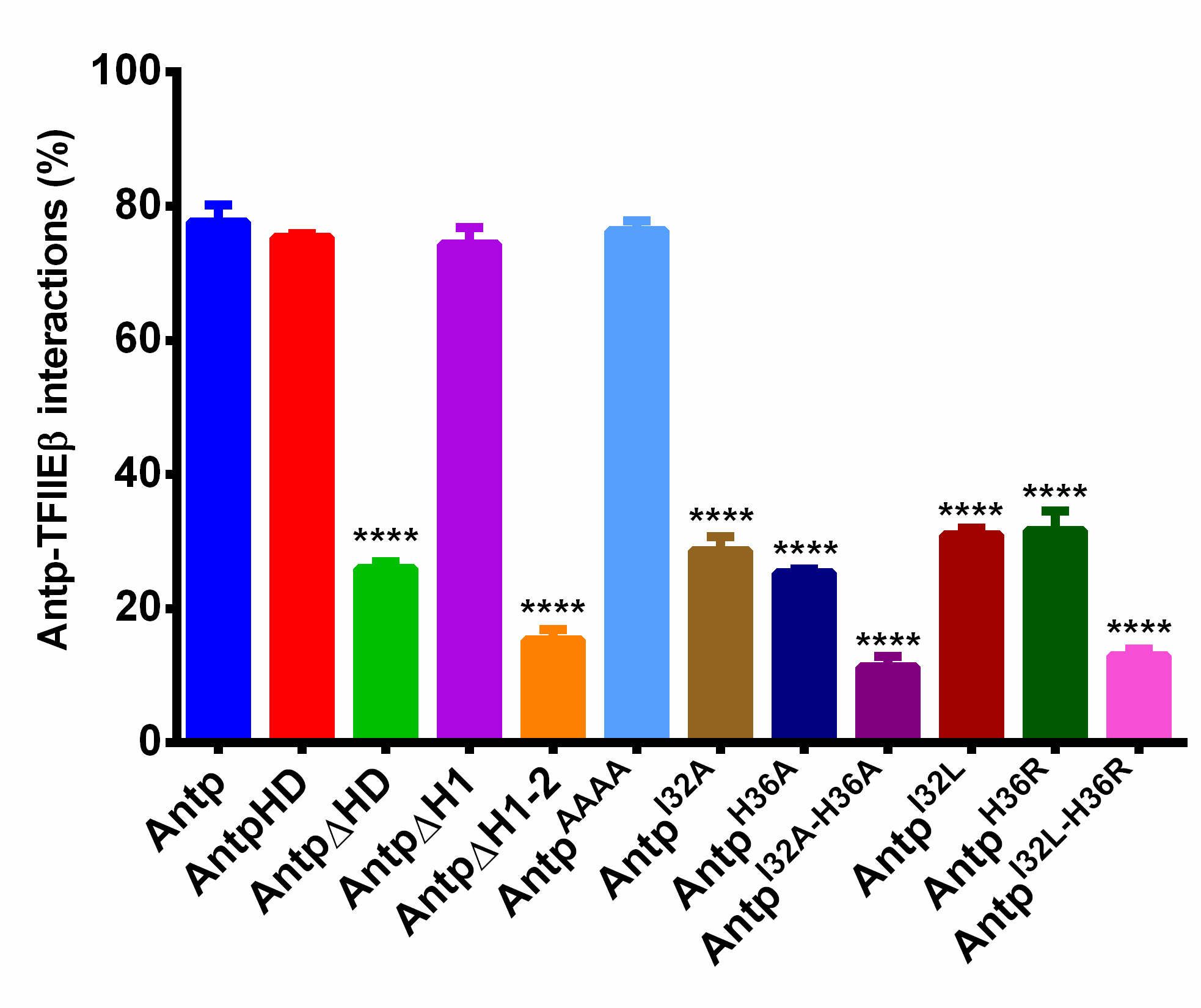

Supplement: S1 Fig — The percentage of the BiFC interactions was analyzed by counting the number of Venus fluorescent cells in one hundred red fluorescent cells (mCherry) of three independent experiments. Statistical quantifications of Antp-TFIIEβ interactions were analyzed using a one-way ANOVA and the post-hoc test Tukey for mean comparison. Antp interaction with TFIIEβ was compared with Antp mutants. AntpHD, AntpΔH1 and AntpAAAA interactions showed no difference compared to Antp, in contrast there is a highly significant difference (****) between Antp and the mutants AntpΔHD, AntpΔH1-2, as well as all helix 2 mutants (p < 0.005). Error bars correspond to standard deviation. (TIF) [file pone.0205905.s002.tif]

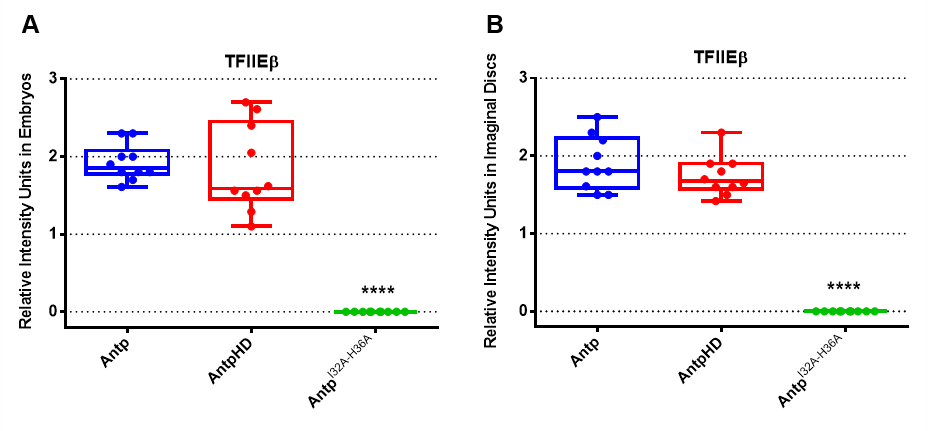

Supplement: S2 Fig — Relative intensity units quantification of BiFC interactions was done using the color histogram function of ImageJ in embryos (A) and in imaginal discs (B). For the statistical analysis we used a one-way ANOVA and the post-hoc test Tukey for mean comparison (p < 0.005). Error bars correspond to standard deviation. (TIF) [file pone.0205905.s003.tif]

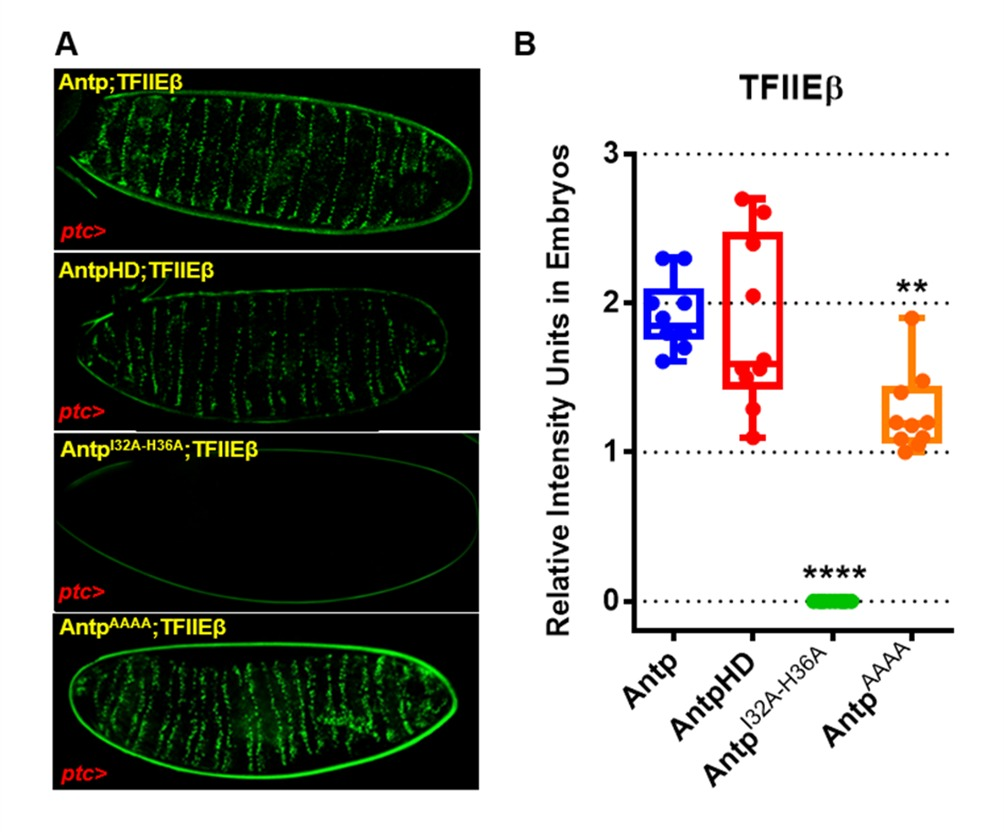

Supplement: S3 Fig — (A) Interactions of Antp, AntpHD and AntpAAAA with TFIIEβ were showed in embryos using BiFC, meanwhile no interaction (BiFC signal) was detected with Antp helix 2 double mutant. (B) Relative intensity units quantification of BiFC interactions in embryos showed significant difference between AntpAAAA interaction with TFIIEβ (**) compared to Antp or AntpHD (p < 0.005). Error bars correspond to standard deviation. (TIF) [file pone.0205905.s004.tif]

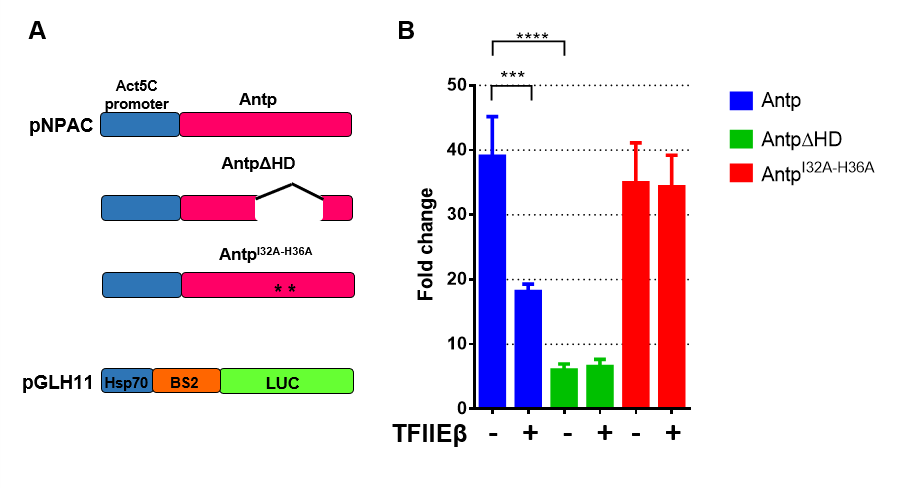

Supplement: S4 Fig — (A) Schematic representation pPAC plasmids containing coding sequences for Antp, AntpΔHD and AntpI32A-H36A directed with Actin5C promoter and luciferase reporter (LUC) containing a minimal Hsp70 promoter and eleven tandem copies of HD-consensus BS2 binding sites. (B) Co-expression of Antp with TFIIEβ resulted in a reduction of 47% (****) of the expression of LUC compared with transactivation of Antp alone (p < 0.005). Mutation of the HD (AntpΔHD) show a 33-fold decrease of transcription (****), whereas the double mutant AntpI32A-H36A activates transcription at the same level of Antp through the BS2 binding sites (p < 0.005). Error bars correspond to standard deviation. (TIF) [file pone.0205905.s005.tif]

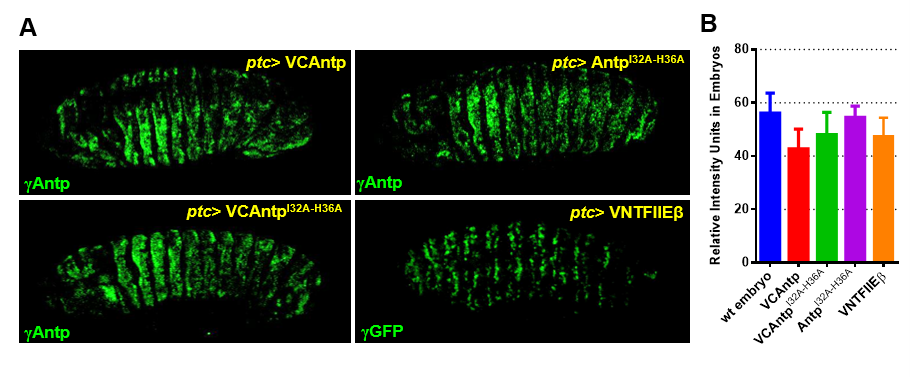

Supplement: S5 Fig — (A) Immunodetection of VCAntp, VCAntpI32A-H36A and AntpI32A-H36A was done using γAntp4C3 and VNTFIIEβ using γ-GFP. (B) Expression level of Antp and TFIIEβ were quantified using the color histogram function of ImageJ and compared to Antp. Relative intensity Units were measured from 5 embryos per construct line using the color histogram function of ImageJ. All lines exhibited expression levels equal or lower than the endogenous Antp protein. Error bars correspond to standard deviation. (TIF) [file pone.0205905.s006.tif]
